# Editorial: The role of dietary fatty acids in metabolic health

**DOI:** 10.3389/fphys.2023.1211151

**Published:** 2023-05-23

**Authors:** Laura Bordoni, Manja Zec, Nenad Naumovski, Domenico Sergi

**Affiliations:** ^1^ Unit of Molecular Biology and Nutrigenomics, School of Pharmacy, University of Camerino, Camerino, Italy; ^2^ School of Nutritional Sciences and Wellness, University of Arizona, Tucson, AZ, United States; ^3^ Discipline of Nutrition and Dietetics, Faculty of Health, University of Canberra, Canberra, ACT, Australia; ^4^ Department of Translational Medicine, University of Ferrara, Ferrara, Italy

**Keywords:** fatty acids, cardiometabolic health, lipid metabolism, lipotoxicity and nutrition, lipoprotein metabolism

Dietary lipid quality plays a pivotal role in shaping cardio-metabolic health (Sears and Perry, 2015; Stonehouse et al., 2021; Sergi et al., 2023). In particular, the ability of different dietary fatty acids to trigger the activation of diverse intracellular signalling pathways is a key discriminant in dictating their impact on cardiometabolic health (Li et al., 2020). Thus, it is now evident that the ability of dietary lipids to modulate health outcomes is not merely driver by their energy density, instead the quality of the dietary lipids assumes a crucial importance to dictate their metabolic impact. The overconsumption of long-chain saturated fatty acids (LCSFA) in concomitance with the reduction in the intake of unsaturated fatty acids (mainly omega-3s), represents one of the major drivers of the metabolic derangements ascribed to the Western diet, namely, obesity and its cardiometabolic complications. Indeed, saturated fatty acids, mainly in the form of LCSFA, have been shown to disrupt whole body metabolic health by negatively affecting key metabolically active tissues including skeletal muscle (Sergi et al., 2021), liver (Luukkonen et al., 2018) as well as the centre for the control of energy balance in the hypothalamus (Valdearcos et al., 2014). In these tissues LCSFA trigger metabolic inflammation, mitochondrial dysfunction and endoplasmic reticulum stress, which, in turn, are key pathogenetic mechanisms responsible for promoting metabolic derangements (Lepretti et al., 2018; Wu and Ballantyne, 2020). Particularly, the effects of LCSFA on metabolic health appear to be underpinned by lipotoxicity which was proposed as a key phenomenon linking lipid metabolism to obesity and its comorbidities (Unger, 2002). Additionally, fatty acid quality is also known to impact upon the circulating lipid profile, in terms of total, low density lipoprotein (LDL) and high density lipoprotein (HDL) cholesterol as well as triglycerides, which, in turn, have a crucial role in cardiovascular health. Thus, this Research Topic aims at gathering novel evidence elucidating the impact of dietary lipids on cardiometabolic health and dissenting the molecular mechanisms underpinning their effects.

In this regard, defects in LDL and HDL-cholesterol metabolism are key in promoting atherosclerosis (Quispe et al., 2020). In this context, Fuller et al. evaluated the effects of high-fat feeding on atherosclerosis in mice lacking either the scavenger receptor class B type I, the LDL receptor or the apolipoprotein E. After 20 weeks on a high-fat diet supplemented with sodium cholate, scavenger receptor class B type I-deficient mice, despite having lower LDL cholesterol level relative to apolipoprotein E, developed a more pronounced degree of atherosclerosis in their descending aortas and coronary arteries. This effect was paralleled by the upregulation of inflammatory responses, marked by the induction of tumour necrosis factor alpha levels as well as lymphocytosis and monocytosis in coronary artery endothelial cells compared to the other mice strains assessed. In light of these findings, the development of atherosclerosis in scavenger receptor class B type I knockout mice is driven by pathogenetic factors which act synergistically with hypercholesterolemia, including inflammation deregulation of the immune system and endothelial defects. Thus, besides the importance of LDL cholesterol in the pathogenesis of atherosclerosis, an impairment in HDL cholesterol metabolism also contributes to the pathogenesis of cardiovascular disease (CVD) (Fuller et al.).

The beneficial effects of seafood consumption on cardiovascular health have been widely described. However, conflicting findings are present in literature about different seafood intake, consumption frequency and long-term effects on human health (Virtanen et al., 2008; Siscovick et al., 2017; Ferrante et al., 2019; Danopoulos et al., 2020; Jayedi and Shab-Bidar, 2020; Mohan et al., 2021). In a prospective study, Critselis et al. investigated the impact of seafood intake on CVD incidence in a period of 10 and 20 years in the ATTICA cohort. Fatal and non-fatal CVD incidence were studied as outcomes and the effect of both total fish intake and omega-3-rich fish intake was evaluated. Dietary habits were assessed based on a validated semi-quantitative food-frequency questionnaire. Of 3,042 initially enrolled participants, 10-year follow-up evaluation was achieved in 2,583 participant and 20-year follow-up was achieved in 2,169 participants. In this study, total seafood intake >2 serving per week was associated to a decreased risk of developing CVD risk of at least 27% at 10 years, and 18% at 20 years. In particular, participants with a higher fish intake had a lower 10-year CVD risk and 76% decreased risk of 10-year CVD mortality. These data further support the necessity for public health intervention promoting the consumption of fish, especially omega-3-rich fish, to reduce the CVD burden on the health system.

Besides seafood, walnuts also contain a cardio-metabolically beneficial fatty acid profile. In keeping with this, Bošković et al., investigated whether a six week walnut supplementation in mice could counter the deleterious effects induced by fructose, particularly with regard to the activation of the cardiac renin-angiotensin system (RAS) and NF-κB. Additionally, authors investigated if walnuts were able to affect the mouse heart lipid profile by inducing a shift in the omega-6/omega-3 PUFA ratio. Walnut supplementation led to an increase in Angiotensin-converting enzyme (ACE) 2 protein level in the heart of mice fed a high fructose diet which occurred in parallel with a drop in the arachidonic acid/eicosapentaenoic acid ratio relative to controls. However, walnut supplementation failed to counter the upregulation of NF-κB protein triggered by the high-fructose diet (Boskovic et al.). In conclusion, walnut supplementation may benefit cardiac health by modulating both the heart fatty acid profile as well as the activation of the RAS.

Always in line with the ability of dietary lipids to trigger intracellular signal transduction pathways, a review article by Son and Paton provides an in-depth summary of the potential mechanisms of action associated with the free fatty acid-induced cell signalling, angiopoietin-like protein 4 (ANGPTL4), and skeletal muscle differentiation. The complex role of free fatty acids (FFA) in skeletal muscles is proposed to be associated with signal transduction, transcriptional regulation, energy production and storage. Nevertheless, higher intake of FFAs is also associated with negative health outcomes such as inflammatory response, insulin resistance and development of metabolic syndrome, as previously discussed. Furthermore, when FFA are provided in excess, they may promote muscle wasting and impaired lipid metabolism (Sieber and Jehle, 2014). The ANGPTL4 is one of the most investigated members of the ANGPTL protein family (Son and Paton). It is expressed in metabolic tissues including the skeletal muscle, adipose tissue and the liver, it participates in number of metabolic processes (angiogenesis, tumorigenesis) and it is associated with the regulation of lipid metabolism, food intake and glucose homeostasis (Cho et al., 2023). Its activity is regulated by several factors (fasting, physical activity, calorie restriction) and current literature supports the notion that FFA can induce the ANGPTL4 expression in skeletal muscle. In this article, based on the available evidence, authors propose that excess long chain FFAs may induce ANGPTL4 expression which, in concert with the inhibition of the Wnt signalling pathway impaired skeletal muscle differentiation (Son and Paton).

Thus, this collection of manuscripts underlines the importance of dietary lipid quality and their metabolism as pivotal discriminants in shaping cardiometabolic outcomes and modulate intracellular signalling.
